# Study of Clinical Characteristics and Cytokine Profiles of Asthmatic Children with Rhinovirus Infection during Acute Asthma Exacerbation at National Hospital of Pediatrics

**DOI:** 10.1155/2018/9375967

**Published:** 2018-08-23

**Authors:** Thuy Nguyen-Thi-Dieu, Huong Le-Thi-Thu, Huong Le-Thi-Minh, An Pham-Nhat, Sy Duong-Quy

**Affiliations:** ^1^Department of Paediatrics, Hanoi University of Medicine, Hanoi, Vietnam; ^2^Department of Immunology, Allergology and Rheumatology, National Hospital of Paediatrics, Hanoi, Vietnam; ^3^Department of Respiratory Diseases, Medical-Biological Research Center, Lam Dong Medical College, Dalat, Vietnam; ^4^Department of Pulmonology and Respiratory Physiology, Cochin Hospital, Paris Descartes University, Paris, France; ^5^Department of Asthma and Immuno-Allergology, Penn State Medical College, Hershey, PA, USA

## Abstract

**Background:**

In children with asthma, the viral infection of airways is usually a main cause of acute asthma exacerbation and hospitalization. However, few studies on clinical and biomolecular characteristics of asthmatic children in this field have been done, especially in emergent countries.

**Objective:**

This study described the clinical and biological characteristics of asthmatic children who had acute asthma exacerbation and rhinovirus (RV) infection.

**Methods:**

Children under 15 years of age hospitalized for acute asthma exacerbation were included. They underwent clinical examination and peripheral blood analyses for the cytokine profile. The severity of acute asthma exacerbation was evaluated by Pediatric Asthma Score (PAS). Healthy children under 15 years of age were also invited in this study.

**Results:**

One hundred fifteen asthmatic children were included in this study. There were 18.2% of mild PAS, 37.4% of moderate PAS, and 44.4% of severe PSA. Among them, 63/115 (54.8%) asthmatic children had positive RV infection (RV^+^). The percentages of asthmatic children with RV^+^ had increased polymorphonuclear leucocytes were significantly higher than asthmatic children with RV^−^. There were no significant differences of the concentrations of non-Th2-related cytokines in asthmatic children with RV^−^ and RV^+^. The concentration of Th2-related cytokines (IL-5 and IL-13) in asthmatic children with RV^+^ was significantly higher than those with RV^−^. However, there was no significant difference for the cytokine profile between mild, moderate, and severe asthma.

**Conclusion:**

RV infection is a main cause of acute asthma exacerbation in children with asthma. The increase of Th2-related cytokines, especially IL-5 and IL-13, is a relevant biomarker for RV infection in asthmatic children with severe exacerbation.

## 1. Introduction

Acute asthma exacerbation (AAE) is usually triggered by viral infection of the airways, particularly by rhinovirus (RV). The role of RV in the onset of AAE has been demonstrated in both children and adults although these patients regularly used preventative treatment for asthma. A previous study showed that 80–85% of AAE in children related to peak flow reduction and wheezing caused by a viral infection in the upper airway tract [1]. RV is also detected in approximately 80% of the wheezing stage in school-age children and 50% in adults.

RV might have a direct effect by switching the inflammation response in the lower respiratory tract, resulting in considerable airway obstruction and clinical symptoms. The impact of RV on the airway of subjects with allergic asthma is mediated by proinflammatory and inflammatory cytokines, inducing the inflammatory process of airways, aggravated asthma severity and AAE. After RV infection, asthmatic patients have increased inflammatory cells located in their airways in response to the inflammatory process. These cells include eosinophils, lymphocytes, and neutrophils. Previous studies also showed that the leukocyte level in bronchoalveolar lavage of asthmatic patients was higher than that of healthy subjects in few days after RV infection [2]. In addition, the disorders of the immunological response, depending on Th2 and Th1, might play an important role in the modulation of the inflammatory process in asthmatic patients with RV.

Until now, although many studies on the role of respiratory viruses in the onset of acute asthma exacerbation in children have been demonstrated, there are few studies on the inflammatory response, particularly on the role of cytokines, in patients with RV-induced AAE. This study described the clinical characteristics and cytokine profiles of asthmatic children with RV infection during AAE.

## 2. Patients and Methods

### 2.1. Patients

Children under 15 years of age with acute asthma exacerbation (AAE) who were hospitalized in the Department of Immunology, Allergology and Rheumatology of National Hospital of Paediatrics, Hanoi, Vietnam, were included in the present study. Parents or legal guardians signed a written consent form for the patient's participation. They had been informed about the study protocol, and the functional and biological testings that could be conducted. This study was approved by the Ethics Committee of the National Hospital of Paediatrics (registered certificate of 954B/BV NTW-VNCSKTE).

### 2.2. Methods

#### 2.2.1. Inclusion Criteria

Children were diagnosed with asthma according to GINA (Global Initiative for Asthma) [3], and they were hospitalized by AAE. The patients and their families agreed to participate in the study as described above.

#### 2.2.2. Diagnosis of Asthma Severity

The diagnosis of asthma was based on international recommendations for children [3]. The severity of AAE was assessed according to the pediatric asthma score (PAS) [4, 5]; the examination consisted of five components: respiratory rate, oxygen requirement, respiratory muscle retractions, auscultation, and dyspnoea; each component was scored from 1 to 3 according to the severity of these symptoms; the total scores ranged from 5 to 7 for mild asthma exacerbation, 8 to 11 for moderate asthma exacerbation, and 12 to 15 for severe asthma exacerbation.

#### 2.2.3. Exclusion Criteria

Asthmatic children suffering from more severe illnesses (hyperthyroidism, arrhythmias, congenital heart diseases, rheumatic heart diseases, etc.) or asthmatic patients hospitalized for acute exacerbation due to other causes (heart failure, pneumothorax, airway obstruction by a foreign body, etc.) were excluded from the present study. Children with AAE who were positive with other viral infections (respiratory syncytial virus or influenza virus) were excluded from the study. Patients or their families who did not agree to participate in the research were also excluded.

#### 2.2.4. Control Subjects

To compare the concentration of cytokines between subjects with AAE and healthy subjects, thirty healthy children under 15 years of age who did not suffer from any acute and chronic diseases and went to the National Hospital of Pediatrics for regular health control were invited to participate in this study as control subjects after receiving the permission from their parents or legal guardians.

#### 2.2.5. Diagnosis of RV Infection

RV infection was diagnosed by real-time polymerase chain reaction (PCR) assays to detect rhinovirus in nasopharyngeal aspirates. The nasopharyngeal aspirates were done for all children on the first day of hospitalization. The laboratory procedure was performed at the Microbiology Department of the National Hospital of Paediatrics.

The nasopharyngeal aspirates were collected by using a sterilized tube passing through the nose into the nasal cavity at the mid distance between the nose and the ear lobe. Five milliliters of nasopharyngeal aspirates was done for each study subject. Nasopharyngeal fluids were analyzed by the CFX96TM real-time system CC3071 (Bio-Rad, Hercules, CA, USA) for diagnosing RV infection.

#### 2.2.6. Quantification of Cytokines in Peripheral Blood

Blood tests of cytokine quantification in serum were done in the Immunology Laboratory of the Military Medical Academy 103 (Hanoi, Vietnam). Cytokine quantification was conducted with the Flow cytometry-assisted immunoassay technology and Bio-Plex system (Bio-Rad, Hercules, CA, USA).

Briefly, venous blood samples (2 mL) from patients and control subjects were drawn into tubes without anticoagulant and transferred directly to the laboratory for analysis. Monoclonal antibodies (Bio-Rad, Hercules, CA, USA) were used for nine cytokines related to type 1 helper T (Th1) cells (IL-2, IL-8, IFN-*γ*, and TNF-*α*), regulatory T cells (IL-10), and type 2 helper T (Th2) cells (GM-CSF, IL-4, IL-5, and IL-13) (Bio-Plex® Human Cytokines, Bio-Rad). The tests were conducted using a flow cytometry-assisted immunoassay provided by the Bio-Plex Multiplex System (Bio-Rad). Blood samples without anticoagulant were placed in an incubator (37°C) for 30 min, then centrifuged at 1,000*g* for 5 min at 4°C (ScanSpeed 416, LaboGene, Lynge, Denmark), and then at 4°C for 15 min at 10,000*g* (ScanSpeed 416, LaboGene) and prepared for freezing. All samples were stored at −80°C until tested.

Cytokines were detected by immunofluorescence using the sandwich technique on the surface of polystyrene microspheres in accordance with the manufacturer's protocol as described previously [6]. Based on the density of the fluorescence emitted from the microspheres incubated with known concentrations of cytokines, the cytokines in the study samples were quantified using Bio-Plex Manager™ Software (Bio-Rad). For each cytokine, the curve fit parameters were established with international cytokine standards and the master curves were used to analyse the immunoassay data.

#### 2.2.7. Statistical Analysis

The statistical analyses were performed with SPSS® statistical package, version 22.0 (SPSS Inc., Chicago, IL, USA) for Windows®. Qualitative variables were expressed as numbers or percentages. Continuous variables were expressed as mean  ±  SD. Normal distribution was tested by using the Skewness-Kurtosis test. Mann–Whitney *U* test was used to evaluate the differences of continuous variables between two groups. *Z*-test was used to compare the frequency between groups. Kruskall–Wallis test was used for multiple comparisons. *P* value  <  0.05 was considered statistically significant.

## 4. Results

### 4.1. Clinical Characteristics of Asthmatic Children

One hundred fifteen asthmatic children were included in this study (Table 1). There were 19.1% of subjects less than 2 years, 47% subjects from 2 to 5 years, and 33.9% subjects more than 5 years of age. Among them, 16.5% study subjects had eczema, 58.3% had allergic rhinitis, and 13.9% had seasonal allergic (Table 1). Almost all study subjects with asthma were living in city than village (89.6% versus 10.4%; Table 1). There were 66% of asthmatic children who had second-hand smoking, and 82% of them had domestic animals (dog or cat). There were 18.2% of study children with mild PAS (pediatric asthma score), 37.4% with moderate PAS, and 44.4% with severe PAS (Table 1).

### 4.2. Clinical and Biological Characteristics of Asthmatic Children Classified by RV Infection

The result showed that there were 63/115 (54.8%) asthmatic children with positive rhinovirus infection (RV^+^) versus 45.2% with negative rhinovirus infection (RV^–^). There was not any significant difference between asthmatic children with RV^+^ versus RV^−^ concerning male-female ratio, atopy, second-hand smoking, and domestic pest exposure (Table 2). The percentage of asthmatic children with RV^+^ was varied by age groups. Children from 2 to 5 years of age had the highest prevalence of RV^+^ in their acute asthma exacerbation (AAE), accounting for 61.1%. Children under 2 years of age had the lowest prevalence of RV^+^, accounting for 36.4% (Table 2).

**Figure 1:**
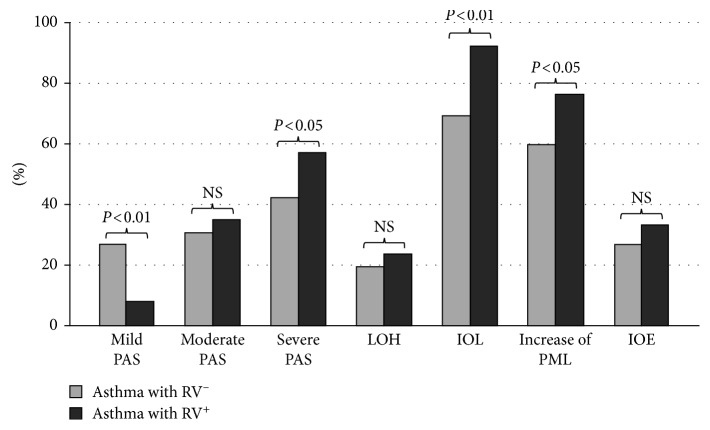
Comparison of clinical and biological characteristics of asthmatic children classified by positive or negative rhinovirus infection. PAS: pediatric asthma scores; LOH: length of hospitalization; IOL: increase of leukocytes; PML: polymorphonuclear leukocytes; IOE: increase of eosinophils.

The percentage of asthmatic children with mild PAS and RV^−^ was significantly higher than that of those with RV^+^ (26.9% versus 7.9%; *P* < 0.01; Table 2; Figure 1). Inversely, the percentage of asthmatic children with severe PAS and RV^+^ was significantly higher than that of those with RV^−^ (57.2% versus 42.4%; *P* < 0.05; Table 2; Figure 1). There was no significant difference in the percentage of length of hospitalization between asthmatic children with RV^+^ versus RV^−^ classified by 1–3 days, >3–5 days, and >5 days (Table 2; Figure 1).

The percentage of asthmatic children with RV^+^ who had increased polymorphonuclear leucocytes was significantly higher than that in asthmatic children with RV^−^ (92.1% and 76.2% versus 69.2% and 59.6%; *P* < 0.01 and *P* < 0.05, resp.; Table 2; Figure 1). There was no significant difference of the percentage of increased and non-increased eosinophils between asthmatic children with RV^+^ or RV^−^ (Table 2; Figure 1).

### 4.3. Modification of Cytokine Profile of Asthmatic Children Classified by RV Infection

**Figure 2:**
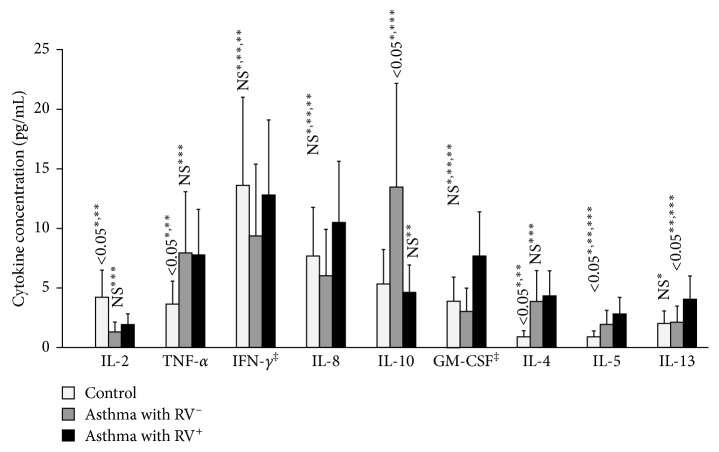
Cytokine profile of study subject classified by control and positive or negative rhinovirus infection. RV: rhinovirus infection. ^*∗*^Asthma with RV^−^ versus control; ^*∗∗*^asthma with RV^+^ versus control; ^*∗∗∗*^asthma with RV^+^ versus asthma with RV^−^; ^‡^multiplication of five.

The result showed that, concerning non-Th2-related cytokines, the concentration of IL-2 in asthmatic children with or without RV infection was significantly lower than that in control subjects (Table 3; Figure 2). There were no significant differences of the concentrations of IL-8 and IFN-*γ* in asthmatic children with RV^−^, RV^+^, and control subjects (Table 3; Figure 2). The concentration of TNF-*α* in asthmatic children with or without RV infection was higher than that in control subjects (*P* < 0.05 and *P* < 0.05, resp.), while there was only IL-10 concentration in AAE subjects with RV^−^ which was significantly higher than that in control and AAE subjects with RV^+^ (Table 3; Figure 2).

Although the concentration of GM-CSF (Th2-related cytokine) was not different between asthmatic children with and without RV infection and control subjects, the concentrations of IL-4 were significantly higher than that in control subjects (4.32 ± 2.56 pg/mL and 3.91 ± 1.30 pg/mL versus 0.86 ± 0.32 pg/mL; *P* < 0.05 and *P* < 0.05, resp.; Table 3; Figure 2). However, there was no significant difference of IL-4 concentration between asthmatic children with RV^+^ and RV^−^ (Table 3; Figure 2). The concentration of IL-5 in asthmatic children with RV^+^ was significantly higher than that in asthmatic children with RV^−^ and in control subjects (2.79 ± 2.73 pg/mL versus 1.85 ± 2.16 pg/mL and 0.89 ± 0.78 pg/mL; *P* < 0.05 and *P* < 0.05, resp.; Table 3; Figure 2). The concentration of IL-13 in asthmatic children with RV^+^ was significantly higher than that in asthmatic children with RV^−^ and in control subjects (3.99 ± 6.46 pg/mL versus 2.05 ±1.51 pg/mL and 2.02 ± 1.92 pg/mL; *P* < 0.05 and *P* < 0.05, resp.; Table 3; Figure 2).

### 4.4. Modification of Cytokine Concentration of Asthmatic Children without or with RV Infection Classified by PAS Severity

There were no significant differences of the concentration of non-Th2-related cytokines (IL-2, IL-8, IL-10, TNF-*α*, and IFN-*γ*) between asthmatic children with RV^−^ having mild, moderate, or severe PAS (Pediatric Asthma Score; Table 4). However, in asthmatic children with RV^+^, the concentration of TNF-*α* in asthmatic children with RV^+^ and severe PAS was significantly higher than that in those with mild and moderate PAS (*P* < 0.05 and *P* < 0.05, resp.; Table 4). Whereas, the concentration of IFN-*γ* in asthmatic children with RV^+^ and moderate or severe PAS was lower than that in mild PAS (Table 4). The concentrations of Th2-related cytokines (GM-CSF, IL-4, IL-5, and IL-13) in asthmatic children (with or without RV infection) were not significantly different between asthmatic children with mild, moderate, and severe PAS (Table 4).

## 5. Discussion

Respiratory viral infection is the leading cause of acute asthma exacerbation in both children and adults. The results of present study showed that 54.8% of study subjects with AAE had the infection with RV infection during the study period. Furthermore, we could observe that RV infection was associated with more severe exacerbation. The RV infection per se did not, however, increase the number of days for hospitalization length. Interestingly, the concentrations of IL-5 and IL-13 were significantly increased in asthmatic children with RV^+^. Especially, asthmatic children with RV+ and severe PAS had high level of TNF-*α* concentration.

A previous study showed that the majority of acute asthma attacks have been triggered by respiratory viruses, in which rhinovirus seems to be the most common [7]. Jackson and Johnston [8] also demonstrated that viral infection had been found in 90% of children during the wheezing stage. This study included 259 children less than 3 years of age with wheezing and showed that there was a strong correlation between respiratory viral infection and the risk of asthma onset. In addition, the authors showed that RV increased the risk of acute asthma exacerbation by 9.8 times. Moreover, in children over 3 years, wheezing associated with RV infection had a risk of asthma onset by 25.6 times higher. Another study involved 142 asthmatic children in the UK including 65 asthmatic children with acute asthma exacerbation and 77 without asthma crisis showed that 60% children with acute asthma exacerbation and 18% without acute asthma exacerbation had RV infection [9]. Mak et al. conducted the isolation of virus in 128 children with acute asthma exacerbation and 192 healthy children in Hongkong, showing that 84.9% of asthmatic children were positive with RV, compared with 33% in the control group [10]. All these results suggest that RV infection might be a main trigger of acute asthma exacerbation.

The present study also showed that, in children with asthma, RV infection increased the percentage of study subjects with severe acute asthma exacerbation (Table 2). However, the length of hospitalization for acute severe asthma exacerbation was not significantly different between asthmatic children with or without RV infection (Table 2; Figure 1). The link between asthma severity and RV infection in asthmatic patients might be due to the impairment of the lung function. By measuring the daily lung function of asthmatic patients with RV infection, Grunberg et al. [11] demonstrated that FEV1 reduced immediately after RV infection, and the reduction was at the maximum after 2 days and correlated with asthma symptoms and bronchial hyperresponsiveness. The increase of lower airway hyperresponsiveness in asthmatic patients with RV infection has been founded in other studies [12]. In the present study, because the majority of study population was less than 5 years, the spirometry could not be done for all the study subjects.

In asthmatic patients, RV infection has been known as the main cause of increasing inflammatory cells during acute asthma exacerbation. Previous studies showed that there was the change of cells in sputum of patients with acute asthma exacerbation having RV infection with a significant increase of polymorphonuclear leukocytes and eosinophils [2, 13]. In the present study, the peripheral blood analysis showed there was a significant increase of polymorphonuclear leukocytes in asthmatic children with acute asthma exacerbation and RV infection in comparison with those without RV infection, and although the percentage of asthmatic children with RV^+^ who had increased eosinophils was higher than that in asthmatic children with RV^−^, there was no significant difference (Table 2 and Figure 1).

Besides its role in airway hypersensitivity, it has been suggested that RV infection might have a potential role for early-onset of asthma and especially for acute asthma exacerbation by the upregulation of cellular and biomolecular inflammatory processes [2, 14]. In the present study, the results of cytokine profile analyses showed that although non-Th2 cytokine concentrations of IL-2, IL-8, TNF-*α*, and IFN-*γ* were not significantly different between asthmatic children with and without RV infection, the concentration of IL-10, however, in subjects with RV infection was significantly lower than those without RV infection (Table 3; Figure 2). This result suggests that the concentration of IL-10 might be decreased in asthmatic children with AAE and viral infection (RV) and increased in AAE due to nonviral infection.

In asthmatic children without RV infection, the concentrations of non-Th2 cytokines (IL-2, IL-8, TNF-*α*, and IFN-*γ*) were not significantly different between mild, moderate, or severe asthma measured by Pediatric Asthma Score (PAS; Table 4).

The results of the present study showed that, among Th2-related cytokine profiles (GM-CSF, IL-4, IL-5, and IL-13), the concentration of GM-CSF was not significantly different between healthy subjects and asthmatic children with or without RV infection (Table 3; Figure 2). It suggests that GM-CSF might have a less important role in acute asthma exacerbation and especially for which had been triggered by RV infection. The concentrations of IL-5 and IL-13 in asthmatic children with RV infection were significantly higher than that in asthmatic children without RV infection (*P* < 0.05; Table 3) while there was no significant difference between them for IL-4 concentration. However, it was significantly higher than that in healthy control. It also suggests that, although IL-4 concentration was increased in asthmatic patients, it was not different in acute asthma exacerbation triggered by or not by RV infection. It has been demonstrated that IL-4 is a major cytokine related to the pathology of allergic inflammatory response. It had an important role in regulating the lymphocytes B producing IgE as well as differentiating Th0 into Th2 [15]. IL-4 increased in blood and bronchoalveolar lavage in patients with allergic asthma, particularly high in patients with severe asthma which was resistant to corticosteroids [16]. IL-4 also increased the bronchial hyperresponsiveness in patients with asthma [17]. In our study, the concentrations of IL-4 had been increased during acute asthma exacerbation, in comparison with that of healthy controls (Table 3 and Figure 2).

The results of present study showed that, although the concentrations of IL-5 and IL-13 were higher in asthmatic children with RV infection, it was not significantly different between mild, moderate, and severe acute asthma exacerbation (Table 4). The role of IL-5 in asthma, especially in asthma with acute exacerbation has been demonstrated. IL-5, known as a cytokine of Th2 cells, plays a key role in indirect inflammation by eosinophils, stimulating the differentiation of eosinophils and maintaining the survival of eosinophils [15]. A previous study revealed that the high concentration of IL-5 in the peripheral blood was also associated with the increase of exacerbation frequency in children with asthma [18]. Currently, IL-13 is considered as a potential pathway for target treatment in allergic asthma. In severe asthma, IL-13 has been known as an inflammatory mediator causing bronchial contraction and hypersecretion, bronchial hyperresponsiveness, and asthma severity [19–22]. However, in the limit of our study, we could not find out the correlation between RV infection and acute exacerbation severity in children with asthma.

## 6. Conclusion

RV infection is a common cause of the acute asthma exacerbation in children with asthma. The clinical and biological characteristics of asthmatic children with RV infection are usually marked by the increase of polymorphonuclear leukocytes and Th2-related cytokines, especially with IL-5 and IL-13. However, more studies with large number of asthmatic children is necessary to clarify the role of RV infection in severe asthma.

## Figures and Tables

**Table 1:** Clinical characteristics of study children with asthma.

Characteristics	Number (*n*=115)	Percentage (%)
Age		
<2 years	22	19.1
2–5 years	54	47.0
>5 years	39	33.9

Sex		
Male/female	71/44	61.7

Atopy		
Eczema	19	16.5
Skin rash	11	9.6
Allergic rhinitis	67	58.3
Seasonal allergy	16	13.9
Others	9	7.8

Living place		
City	103	89.6
Village	12	10.4

Second-hand smoking	66	57.4

Domestic animals^*∗*^	82	71.3

PAS		
Mild	21	18.2
Moderate	43	37.4
Severe	51	44.4

^*∗*^Dog or cat. PAS: pediatric asthma scores consisted of five components: (i) respiratory rate, (ii) oxygen requirement, (iii) respiratory muscle retractions, (iv) auscultation, and (v) dyspnea; each component was scored from 1 to 3 according to the severity of the symptoms as follows: (i) respiratory rate: ≤34 (2-3 years), ≤30 (4-5 years), ≤26 (6–12 years), ≤23 (>12 years)  =  1; 35–39 (2-3 years), 31–35 (4-5 years), 27–30 (6–12 years), ≤24–27 (>12 years)  =  2; ≥40 (2–3 years), ≥36 (4–5 years), ≥31 (6–12 years), ≥28 (>12 years)  =  3; (ii) oxygen requirements (peripheral capillary oxygen saturation [SpO_2_]): >95% on room air  =  1; 90–95% on room air  =  2; <90% on room air or on any oxygen  =  3; (iii) respiratory muscle retractions: none or intercostal  =  1; intercostal and substernal  =  2; intercostal, substernal, and supraclavicular  =  3; (iv) auscultation: normal breath sounds to end-expiratory wheeze only  =  1; expiratory wheezing  =  2; inspiratory and expiratory wheezing  =  3; (v) dyspnoea: speaks in sentences  =  1; speaks in partial or short sentences  =  2; speaks in single words/short phrases/grunting  =  3. The total scores ranged from 5–7 for mild asthma exacerbation, 8–11 for moderate asthma exacerbation, and 12–15 for severe asthma exacerbation.

**Table 2:** Clinical and biological characteristics of asthmatic children classified by RV infection.

Characteristics	Asthma with RV^−^	Asthma with RV^+^	*P*
Subjects, *N* (%)	52 (45.2)	63 (54.8)	NS

Age			
<2 years, *N* (%)	13 (25.0)	11 (17.5)	NS
Mean ± SD (months)	16 ± 4	18 ± 5	NS
2–5 years, *N* (%)	19 (36.5)	33 (52.4)	NS
Mean ± SD (months)	41 ± 12	42 ± 13	NS
>5 years, *N* (%)	20 (38.5)	19 (30.2)	NS
Mean ± SD (months)	95 ± 20	110 ± 31	NS

Male/female (ratio)	2.0	1.7	NS

Atopy			
Eczema (%)	47.5	52.5	NS
Allergic rhinitis (%)	62.0	70.9	NS
Seasonal allergy (%)	95.9	94.8	NS
Others, *N* (%)	8.1	14.8	NS

Second-hand smoking (%)	18.3	17.2	NS

Domestic animals^†^ (%)	4.1	3.6	NS

PAS			
Mild, *N* (%)	14 (26.9)	5 (7.9)	<0.01
Moderate, *N* (%)	16 (30.7)	22 (34.9)	NS
Severe, *N* (%)	22 (42.4)	36 (57.2)	<0.05

Length of hospitalization			
1–3 days, *N* (%)	14 (27.5)	21 (33.3)	NS
Mean ± SD (days)	2.8 ± 0.4	2.7 ± 0.6	NS
>3–5 days, *N* (%)	27 (52.9)	27 (42.8)	NS
Mean ± SD (days)	4.4 ± 0.5	4.5 ± 0.5	NS
>5 days, *N* (%)	10 (19.6)	15 (23.8)	NS
Mean ± SD (days)	8.0 ± 2.0	7.4 ± 1.9	NS

Leukocytes			
Increasing, *N* (%)	36 (69.2)	58 (92.1)	<0.01
Mean ± SD	16023 ± 4532	15822 ± 4517	NS
** **Not increasing, *N* (%)	16 (30.8)	5 (7.9)	<0.01
Mean ± SD	7518 ± 1716	6146 ± 3867	NS

Polymorphonuclear leukocytes			
Increasing, *N* (%)	31 (59.6)	48 (76.2)	<0.05
Percentage ± SD	67.6 ± 12.8	72.2 ± 12.4	NS
Not increasing, *N* (%)	21 (40.4)	15 (23.8)	<0.05
Percentage ± SD	43.8 ± 11.1	42.3 ± 15.5	NS

Eosinophils			
Increasing, *N* (%)	14 (26.9)	20 (33.3)	NS
Percentage ± SD	7.7 ± 3.4	6.3 ± 2.6	NS
Not increasing, *N* (%)	38 (73.1)	40 (66.7)	NS
Percentage ± SD	1.2 ± 1.1	1.2 ± 1.1	NS

^†^Dog or cat. RV: rhinovirus; RV^+^: positive RV infection; RV^−^: negative RV infection. PAS: pediatric asthma score consisted of five components as described in footnote of Table 1.

**Table 3:** Comparison of cytokine concentration of study subjects classified by positive or negative rhinovirus infection versus control.

Cytokines (pg/mL)	Control (*N*=30)	Asthma with RV^−^ (*N*=52)	Asthma with RV^+^ (*N*=63)	*P* ^*∗∗*^
Non-Th2-related cytokines				
IL-2	4.21 ± 2.88	1.27 ± 2.43	1.86 ± 3.17	<0.05^*∗*^^,^^*∗∗*^, NS^*∗∗∗*^
IL-8	7.58 ± 7.40	6.02 ± 5.65	10.37 ± 15.42	NS^*∗*^^,^^*∗∗*^^,^^*∗∗∗*^
IL-10	5.28 ± 9.78	13.44 ± 16.52	4.57 ± 6.87	<0.05^*∗*^^,^^*∗∗∗*^; NS^*∗∗*^
TNF-*α*	3.61 ± 8.46	7.94 ± 37.02	7.65 ± 32.13	<0.05^*∗*^^,^^*∗∗*^, NS^*∗∗∗*^
IFN-*γ*	77.56 ± 66.43	46.47 ± 68.13	63.50 ± 53.94	NS^*∗*^^,^^*∗∗*^^,^^*∗∗∗*^

Th2-related cytokines				
GM-CSF	18.93 ± 48.54	14.70 ± 29.46	38.04 ± 44.79	NS^*∗*^^,^^*∗∗*^^,^^*∗∗∗*^
IL-4	0.86 ± 0.32	3.91 ± 1.30	4.32 ± 2.56	<0.05^*∗*^^,^^*∗∗*^; NS^*∗∗∗*^
IL-5	0.89 ± 0.78	1.85 ± 2.16	2.79 ± 2.73	<0.05^*∗*^^,^^*∗∗*^^,^^*∗∗∗*^
IL-13	2.02 ± 1.92	2.05 ± 1.51	3.99 ± 6.46	NS^*∗*^; <0.05^*∗∗*^^,^^*∗∗∗*^

RV: rhinovirus; RV^+^: positive RV infection; RV^−^: negative RV infection. ^*∗*^Asthma with RV^−^ versus control; ^*∗∗*^asthma with RV^+^ versus control; ^*∗∗∗*^asthma with RV^+^ versus asthma with RV^−^.

**Table 4:** Cytokine profile classified by RV infection and asthma severity.

PAS	Asthma with RV^−^			*P*	Asthma with RV^+^			*P*
	Mild	Moderate	Severe		Mild	Moderate	Severe	
Non-Th2-related cytokines								
IL-2	0.68 ± 0.84	3.13 ± 10.29	0.59 ± 1.09	NS^*∗*^^,^^*∗∗*^^,^^*∗∗∗*^	3.65 ± 6.80	2.12 ± 8.92	1.76 ± 3.71	NS^*∗*^^,^^*∗∗*^^,^^*∗∗∗*^
IL-8	7.11 ± 8.35	3.96 ± 1.82	5.24 ± 4.11	NS^*∗*^^,^^*∗∗*^^,^^*∗∗∗*^	8.96 ± 6.39	8.31 ± 4.63	13.86 ± 22.84	NS^*∗*^^,^^*∗∗*^^,^^*∗∗∗*^
IL-10	9.36 ± 21.75	34.07 ± 105.7	3.89 ± 37.20	NS^*∗*^^,^^*∗∗*^^,^^*∗∗∗*^	4.94 ± 2.74	3.82 ± 4.28	5.52 ± 9.48	NS^*∗*^^,^^*∗∗*^^,^^*∗∗∗*^
TNF-*α*	9.01 ± 26.11	18.90 ± 66.49	10.06 ± 1.10	NS^*∗*^^,^^*∗∗*^^,^^*∗∗∗*^	1.68 ± 2.28	1.81 ± 3.20	15.44 ± 38.38	NS^*∗*^; <0.05^*∗∗*^^,^^*∗∗∗*^
IFN-*γ*	37.49 ± 51.02	81.32 ± 168.6	36.98 ± 53.87	NS^*∗*^^,^^*∗∗*^^,^^*∗∗∗*^	122.25 ± 226.1	48.15 ± 76.70	65.78 ± 202.1	<0.05^*∗*^^,^^*∗∗*^; NS^*∗∗∗*^

Th2-related cytokines								
GM-CSF	18.15 ± 32.08	19.47 ± 40.37	12.59 ± 21.46	NS^*∗*^^,^^*∗∗*^^,^^*∗∗∗*^	102.38 ± 104.6	24.67 ± 44.35	46.59 ± 148.0	NS^*∗*^^,^^*∗∗*^^,^^*∗∗∗*^
IL-4	4.24 ± 5.12	4.82 ± 6.34	3.93 ± 8.23	NS^*∗*^^,^^*∗∗*^^,^^*∗∗∗*^	3.03 ± 6,65	4,18 ± 8,23	4,23 ± 3,56	NS^*∗*^^,^^*∗∗*^^,^^*∗∗∗*^
IL-5	1.66 ± 1.40	2.50 ± 2.57	1.98 ± 2.53	NS^*∗*^^,^^*∗∗*^^,^^*∗∗∗*^	4.42 ± 5.29	2.04 ± 1.89	2.48 ± 2.96	NS^*∗*^^,^^*∗∗*^^,^^*∗∗∗*^
IL-13	2.04 ± 1.54	2.58 ± 1.64	1.83 ± 1.62	NS^*∗*^^,^^*∗∗*^^,^^*∗∗∗*^	2.86 ± 2.84	2.94 ± 28.20	5.11 ± 9.62	NS^*∗*^^,^^*∗∗*^^,^^*∗∗∗*^

RV: rhinovirus; RV^+^: positive RV infection; RV^−^: negative RV infection. ^*∗*^Moderate PAS versus mild PAS; ^*∗∗*^severe PAS versus mild PAS; ^*∗∗∗*^severe PAS versus moderate PAS. PAS: pediatric asthma scores consisted of five components as described in footnote of Table 1.

## Data Availability

The data used to support the findings of this study are available from the corresponding author upon request.
